# How to support children to develop and express their coping preferences around minor invasive medical procedures: children’s and parents’ perspectives

**DOI:** 10.1007/s00431-023-05222-7

**Published:** 2023-10-03

**Authors:** Elisabeth W. Segers, Marjolijn Ketelaar, Marjorie A. C. P. de Man, Lisette Schoonhoven, Elise M. van de Putte, Agnes van den Hoogen

**Affiliations:** 1 Department of Pediatrics, Wilhelmina Children’s Hospital, University Medical Center Utrecht, PO Box 85090, 3508 AB Utrecht, The Netherlands; 2 Center of Excellence for Rehabilitation Medicine, Brain Center University Medical Center Utrecht and De Hoogstraat Rehabilitation, Utrecht, The Netherlands; 3 Nursing Science, Julius Center for Health Sciences and Primary Care, University Medical Center Utrecht, Utrecht University, Utrecht, The Netherlands; 4 School of Health Sciences, Faculty of Environmental and Life Sciences, University of Southampton, Southampton, UK; 5 Department of Neonatology, Wilhelmina Children’s Hospital, University Medical Centre Utrecht, PO Box 85090, 3508 AB Utrecht, The Netherlands

**Keywords:** Pain, Distress, Children, Coping preferences, Empowerment, Medical procedures

## Abstract

Invasive medical procedures in hospitals are major sources of stress in children, causing pain and fear. Non-pharmacological interventions are indispensable in effective pain and fear management. However, these interventions must be personalized to be effective. This qualitative study aims to gain insight into children’s and parents’ experiences, needs, and wishes related to supporting children to develop and express their coping preferences for dealing with pain and fear during minor invasive medical procedures in order to decrease pain and fear. A qualitative study using thematic analysis was performed. Data were collected through semi-structured interviews with children and parents who had undergone at least five minor invasive medical procedures in the last year. Nineteen children (8–18 years) and fourteen parents were interviewed individually. The experiences, needs, and wishes expressed in the interviews could be classified into one overarching theme, that of the personal process, and two content-related sub-themes: feeling trust and gaining control. The personal process was divided into two different phases, that of developing and of expressing coping preferences. Children and parents both reported it as a continuous process, different for every child, with their own unique needs. Children and parents expected personalized attention and tailored support from professionals.

*     Conclusion*: Professionals must combine clinical skills with child-tailored care. In the process of searching for and communicating about coping preferences, children’s unique needs and personal boundaries will thereby be respected. This gives children and parents increased trust and control during invasive medical procedures.

**Table:** 

**What is Known:** *• Untreated pain and stress caused by medical procedures can have severe and important short- and long-term consequences for children. Personalized non-pharmacological interventions are an essential element of procedural pain management.*	
**What is New:** *• A personalized coping strategy is important for children when undergoing medical procedures. Each individual child has a personal way of expressing their own coping strategy. Children and their parents need information and the space to develop and express their individual coping preferences.* *• Children and parents expect to receive child-tailored care from professionals including respect for their own, unique needs and boundaries. Professionals should build trustful relationships and provide appropriately tailored autonomy around medical procedures.*

**What is Known:**

*• Untreated pain and stress caused by medical procedures can have severe and important short- and long-term consequences for children. Personalized non-pharmacological interventions are an essential element of procedural pain management.*

**What is New:**

*• A personalized coping strategy is important for children when undergoing medical procedures. Each individual child has a personal way of expressing their own coping strategy. Children and their parents need information and the space to develop and express their individual coping preferences.*

*• Children and parents expect to receive child-tailored care from professionals including respect for their own, unique needs and boundaries. Professionals should build trustful relationships and provide appropriately tailored autonomy around medical procedures.*

## Introduction

During medical treatment, children are often subject to invasive medical procedures such as cannula insertion. These are major sources of stress in children, causing pain and fear [1–4]. Children who frequently experience these negative feelings are at risk for both short- and long-term consequences on their mental and physical health, leading to increased procedure-related pain, a lower sense of control over their well-being, and ongoing posttraumatic stress responses [5–8]. Therefore, an effective approach minimizing pain and fear is paramount for children who frequently undergo invasive medical procedures.

Non-pharmacological interventions (NPIs), besides pharmacological interventions such as sedation or local anesthesia, are crucial to pain and fear management [9–11]. These interventions distract the focus of attention from the procedure, thereby reducing pain, distress, and anxiety [10]. Many NPIs are simple and require minimal training, and hence can be implemented directly by children and parents [11]. They capitalize on the self-identified strategies children and parents often develop for dealing with pain and fear when they regularly experience medical procedures [12, 13]. However, these interventions are not routinely administered in clinical practice, particularly in the case of minor invasive medical procedures such as intravenous cannulation or nasogastric tube insertion [2, 3, 14].

Several reviews highlight the evidence-based efficacy of different NPIs, but also emphasize the challenges in determining the effectiveness for an individual patient in practice [10, 11, 15, 16]. There is a wide variety in NPIs, which can be applied in many different ways by parents and professionals. In addition, the child’s age and development, temperament, social skills, and previous experiences play an important role. This implies that effectiveness may at least partially depend on the preferences and circumstances of the child and their parents [5, 10, 16–18].

Research into children’s personal preferences during medical procedures is limited. Qualitative studies reveal that children and parents need to get personalized support and information about helpful strategies they can use [19, 20]. In addition, it is important that their wishes and personal boundaries are taken into account [12, 21]. Therefore, a deeper understanding of how to identify children’s individual preferences can help professionals provide personalized strategies.

This qualitative study aims to gain insight into children’s and parents’ experiences, needs, and wishes, related to supporting children to develop and express their coping preferences for dealing with pain and fear during minor invasive medical procedures in order to decrease pain and fear.

## Methods

### Design

A qualitative study was conducted, to enable an in-depth understanding of the experiences, needs, and wishes of both children and parents with regard to supporting children to express their preferences during minor invasive medical procedures. Data were collected through semi-structured interviews with children and parents of a child who had recently undergone invasive medical procedures.

### Study setting

The study was conducted at the Wilhelmina Children Hospital, a tertiary care pediatric university hospital in the Netherlands. Children and parents were recruited from December 2020 to May 2021.

### Ethical approval

The questionnaire and methodology for this study was approved by the Medical Research Ethics committee of the University Medical Centre in Utrecht, Protocol number 20–634/C. The Medical Research Ethics Committee of the UMC Utrecht confirmed that the Dutch Medical Research Involving Human Subjects Act (WMO) did not apply to this study. The study was conducted in accordance with the Declaration of Helsinki.

If children and parents agreed to participate, an assent was obtained from children under twelve and written consent from children from 12 years and older. Parental consent was sought for participating children under 16 years of age and for their own participation. All participants gave written informed consent.

### Inclusion criteria

In order to be eligible to participate in this study, children and parents had to meet all of the following criteria:

- Children had to be between 8 and 18 years old and have experienced at least five minor invasive medical procedures in the last 2 years with three in the last year in a hospital. Or: parents had to have a child (0–18) who had experienced at least five minor invasive medical procedures in the last 2 years with three in the last year in a hospital.
- The medical procedures had to be minor but invasive (e.g. insertion of intravenous cannulas, nasogastric tubes, or urinary catheters), and carried out without sedation.
- Able to speak, write and read Dutch.

### Sampling strategy and data collection

A purposeful sample of children and parents of children who met the inclusion criteria with various health conditions, ages, sex, and different experiences of minor medical procedures was recruited. The researcher (EWS) regularly consulted with the admission planner and nurses on the wards about which children and parents could be approached for this purpose. This purposeful sampling technique was used to ensure in-depth understanding of the experiences.

After permission, EWS approached interested participants for written informed consent. Eligible children and parents were recruited from both an outpatient department and from clinical departments. A letter with general information about the study was sent to eligible parents and children from the outpatient department to give them time to consider participation during treatment.

The semi-structured face-to-face interviews were mostly held in the hospital during admission and took place at the child’s bedside in their own room. Some parents preferred an interview by telephone after discharge from the hospital. When both child and parent wanted to participate, the interviews were conducted separately. Field notes were made after each interview. All interviews were performed by the researcher (EWS), a researcher and practicing nurse, trained in performing interviews and qualitative research. The interviews were structured around the following themes: experiences of medical procedures, how helping strategies were developed, and how to express them. See Appendix: the interview guide for both children and parents. Prior to participant recruitment, the interview guide was pilot tested with two members of the hospital children’s council. All interviews were audio recorded and transcribed verbatim.

### Data analysis

The analysis incorporated reflexive thematic examination of interview data to explore children’s and parents’ feelings and opinions about what helps children during medical procedures.

A six-stage inductive thematic analysis approach, as described by Braun and Clarke, was used to identify and code patterns or themes within and across the interview data [22, 23]. In the first phase, transcripts of the interviews were read and reread. This generated initial codes across the data. Themes were searched for and identified in the second and third phases. This initial analysis was conducted independently and manually by two members of the research team (EWS, AH). Detailed analysis and interpretation resulted in the identification of themes and patterns across questions. Discrepancies between the researchers about how to code the data were resolved through consensus achieved by discussion with a third researcher (MK).

In the fourth phase, after sixteen interviews with the children and fourteen interviews with parents, no additional codes related to the themes were identified in the subsequent interviews, and enrollment stopped [24]. Codes and theme codes were then compared and discussed with the whole research group in the fifth phase. In the final phase, the report was written according to the consolidate criteria for reporting qualitative research (COREQ) guidelines [25].

The qualitative data analysis software program NVivo 11 (QSR International; Melbourne, Australia) was used for data management during the analysis.

## Results

A total of 27 children and 18 parents were approached by nurses to participate in this study. Nineteen children and fourteen parents (two fathers and twelve mothers) were interviewed. In six cases, we interviewed both the child and the parent of the same family. All children and parents were interviewed separately. The interviews with children lasted between 20–35 min, while parent interviews took 20–50 min. Eight children and four parents decided not to participate.

All participating children had experiences with needle-related medical procedures, some of them frequently, and some of the children were familiar with nasogastric tube insertions in the past. The children were aged 8 to 18 years, eleven of them were girls, eight of them were boys. For further child and parent characteristics, see Tables 1 and 2. During the interviews, children and parents primarily shared their experiences of undergoing medical procedures and applying their preferred coping strategy. Children could not always verbalize what they needed to express their coping preferences, even when questioning further.

**Table 1:** Child characteristics

	**Number (*N*)**	**Percentage (%)**
**Gender**		
** Male**	11	58
** Female**	8	42
**Age**		
** 8–12**	5	26
** 13–16**	7	37
** 17–18**	7	37
**Diagnosis**		
** Immunological disease**	4	21
** Inflammatory bowel disease**	4	21
** Juvenile idiopathic arthritis**	2	10
** Other auto immune disease**	2	10
** Neuromuscular disease**	2	10
** Cardiological disease**	2	10
** Hydronephrosis**	1	5
** Cystic fibrosis**	1	5
** Spina bifida**	1	5
**Experienced medical procedures (frequency)**		
** Intravenous blood sample**	19 (> 5 ×)	100
** Intravenous infusion**	19 (> 5 ×)	100
** Gastro nasal tube insertion**	6 (> 3)	32
** Subcutaneous injection**	1 (> 5 ×)	5
** Lumbar puncture**	1 (4 ×)	5

**Table 2:** Parent characteristics

	**Number (*n*)**	**Percentage (%)**
**Gender**		
** Male**	2	14
** Female**	12	86
**Age:**		
** 30–40**	8	57
** 41–55**	6	43
**Educational level**		
** MBO**	6	43
** HBO**	6	43
** University**	2	14
**Gender child**		
** Male**	10	72
** Female**	4	28
**Age child**		
** 0–8**	4	28
** 8–12**	7	50
** 13–16**	2	14
** 17–18**	1	7
**Diagnosis child**		
** Immunologic disease**	4	28
** Nephrotic syndrome**	1	7
** JIA**	1	7
** Spina bifida**	1	7
** IBD**	3	21
** Severe allergic asthma**	1	7
** Metabolic disease**	1	7
** Rare genetic syndrome**	1	7
**Experiences medical procedures child**		
** Intra venous blood sample**	14 (> 5 ×)	100
** Intravenous infusion**	14 (> 5 ×)	100
** Gastro nasal tube insertion**	6 (> 4)	43
** Gastrostomy catheter insertion**	2 (> 5 ×)	14
** Subcutaneous injection**	1 (> 5 ×)	7

From the interviews, one overarching theme was identified: “a personal process”. Within this personal process, two content-related sub-themes were important for both children and parents: feeling trust and gaining control. In addition, two phases could be distinguished in this personal process which could alternate during the process: developing and expressing (own) coping preferences. In every phase, each individual child and parent had their own needs.

### A personal process; finding your own way

The interviews revealed that, when coping with medical procedures and expressing a preferred coping strategy, the children each went through their own individual process. Children of the same age experienced the same medical procedures very differently. They therefore differed in the use of coping strategies and had varied preferences for NPIs. Some children used their preferred coping strategy for a long period of time; others changed their preferred coping strategy regularly—even during one medical procedure—depending on the efficacy of the coping strategy of each child. Hence, there were large variations both in experiences of medical procedures and in coping preferences.

As a result of these varied experiences and preferences, children displayed different needs for help in coping with the pain and stress caused by the medical procedure (Table 3, quote 1a). Some children developed a coping strategy by themselves or with the help of their parents; others needed support from professionals when they underwent medical procedures. Sometimes, a fixed order of the actions during the medical procedure was important.

**Table 3:** Quotes that illustrates the themes

**(Sub) theme/summary**	**Quote**
**(1) Overall theme: a personal process, finding your own way**	
** 1a Different needs for help**	*Child (C)19 (14 yrs) (Interviewer: and when they ask you how do you want it?) Yeah I don’t think I need that either, but…I do understand that the kids like it. So…if they ask, it could be nice. Yes, I think it would be nice anyway*
** 1b Different, unique preferences**	*Parent (P)14 (C 6yrs) Yes, I think the most important thing is that you must never lose sight of the fact that this is different for each child and that it also changes continuously. And tha’s why it’s so important to involve parents and children themselves*
**(2) Sub-theme: to gain control**	
** 2a Coping strategy is important for every child**	*C3 (16 yrs) Yeah, I’ve just really gotten pretty used to it* *I usually look away. That’s what I do. And I try to take my mind somewhere else for a while*
** 2b Professionals must take preferences into account**	*P8 (C 11 yrs) We really have to follow that* [puncture plan]*. So I take that puncture plan into the room every time; I show it, the nurse reads it. ….. I have learned not to wait for the nurse to ask something. No, you just have to say it yourself, ‘this is the plan’. Yeah, that just helps* *C6 (17yrs) If they don’t ask* [my coping preferences], *then I don’t give consent. Then they are not allowed to do it*
** 2c: Many children want to say the coping strategy by themselves**	*C6 (17 yrs)I hope they know, but I say it just to be sure. And if they don’t know, I’ll say it* *C5 (13 yrs) Most of the time, I’ve already told them* [my coping preferences]*. Then I’m sure they know*
**(3) Sub-theme: to feel trust**	
** 3a. The professional knows your coping strategy by forehand**	*C16 (12 yrs): They usually just read the record beforehand. Yes, I want that they just know it what I want…. Yes, that’s actually convenient, then I don’t have to repeat it all…..ehm, I think it also makes me a little calmer*
** 3b. The professional knows you and your life circumstances**	*C9 (17yrs): Yes. You know everyone and everyone knows you. That is…for example, last month I got my driver’s license and then you hear from here: ‘how did it go?’ and things like that. You can just have fun talking and doing it. It give a feeling of peace, actually. Just that you, yes, you can say everything. …… Well just…and if you really don’t like it anymore or something, then you can say it more easily than if you were with someone else. Of course they all do the same things, but still you get a sense of trust or something like that*
** 3c. The professional keeps appointments and respects borders**	*P8 (C 11 yrs) But it’s just very calming that you have a plan in your hands and that you can show it, like: ‘that’s the way it should be’ and here we are walking…I find it very…here everyone is cooperative right away…they get it right away and they stick completely to the plan* *P1 (C 16 yrs) Well, I think that if there was more listening… For example, he already indicated that if he was injected at a certain place in his abdomen, he would experience extra pain. And he also indicated that, but then they said: ‘yes, but, last week you received your shot there so now we have to do it here’ and then I think: ‘yes, you can also try to discuss it’……. Yes and this is still so deep in X’s mind, even after all these years, because he wanted to make sure that he didn’t get the same home-care nurse he had had in that period*
** 3d. The professional is competent**	*P3 (C11yrs): …Because she was in hospital when she was admitted, she was admitted a couple of times when she was really doing badly. Yes, they just did very bad iv cannulations. And it was just an inexperienced nurse who did it with all good intentions. But if you already have a child that perhaps has a certain fear, then it is important that it goes really well, you know*
**(4) Phases in the process of expressing coping preferences**	
** 4a Process: space to discover your own coping strategy**	*C7 (11 yrs): I often used new methods. And finally a method worked and I chose that method* *C15 (10 yrs): Um, well other times I had… when I came here, I had a blood shot and then there was a robot. But now…I hold mommy’s hand …and when they’re poking, mommy and I do a contest, who finds the most figures in the smiley book, who wins. This is better* *C16 (12 yrs) [a tip for others]: Find your own way*
** 4b Process: how to express a coping strategy**	*P2 (C 3yrs): I think that if, before calling X in, they have already read: ‘Oh, he often wants it like this and like that’, then you can both act accordingly without having to discuss this at length. That seems to me to be the advantage of a digital tool and they are able to see it in advance*

Because of children’s different requirements for help, there were also different needs in expressing coping preferences. When children did not need the professional’s assistance during the execution of their coping strategy, they did not feel the necessity of expressing their coping wishes to the professional and had never thought to ask for help. In contrast, when children needed the professionals’ support to execute their coping strategy, it was important for them to express their preferences. For most of the children, it was comfortable if the professional already knew their preferences before the procedure. However, some children wished to express it themselves. More details of the experiences and needs in coping strategy regarding medical procedures are described further below in this results section.

Parents’ experiences resembled those of the child, the reason not to describe these separately. They narrated more elaborately about all the experiences of their child with medical procedures and their unique needs. Although children sometimes could not remember their coping strategies or the needs they have had in the past, parents could give an overview of everything that had happened. When there were problems in the past, they emphasized the need that the professional should view and approach the child as unique. Parents realized the importance of the medical procedure, but emphasized the significance of taking into account their child’s preferences and coping strategies. (Table 3, quote 1b).

### Control and trust

Despite all these differences, the experiences, needs, and wishes about coping preferences narrated in the interviews could be distinguished into two themes: feeling trust and gaining control. Some children and parents seemed to be more in need of control; others emphasized the importance of trust in the professional and how to achieve that.

In each interview, with both children and parents, one or both of these themes were mentioned.

### To gain control

Although children perceived the medical procedures they had to undergo differently, they all experienced stress to a greater or lesser degree. All children had developed a coping strategy to gain control. Some teenagers had had a considerable amount of experience in medical procedures and reported that, although they were used to them, they also used a specific, usually minor, ritual as a coping mechanism, e.g. tapping with their foot or actively guiding their thoughts to other subjects.

Both children and parents stated that a coping strategy is important for them. It provided feelings of control in a situation that was perceived as stressful because it could potentially be painful (Table 3, quote 2a).

When children had developed their coping strategy themselves, they emphasized the importance of professionals taking the preferences and coping strategy into account and acting accordingly (Table 3, quotes 2b). Parents were more aware of the importance of the ritual than children, but some older teenagers with a lot of experience were adamant about their ritual needing to be done in the way they were accustomed to. For children who had previously been very anxious about a procedure, it was especially important that their coping strategy was known and taken into account.

Some children needed to express the coping strategy themselves just before the procedure (Table 3, quotes 2c). They knew what they wanted, and they had experience dealing with many different medical professionals. By emphasizing their coping preferences just before the start of the procedure, they gained a sense of security (control). These were teenagers who had undergone many procedures. They seemed secure about their coping strategy, and they needed the professional when they conducted their personal coping ritual.

### To feel trust

Children and parents reported that they experienced less stress during the medical procedure when there was a feeling of trust in the professionals who were participating in the medical procedure. Several ways to foster feelings of trust around a medical procedure were mentioned, as well as unpleasant events that eliminated trust and increased stress.

The first way to gain trust in the professional is if professionals can show that they are aware of coping preferences (Table 3, quote 3a). Especially younger children did not appear willing to communicate their wishes every time before a procedure. It was difficult for them to tell the reason why, but parents revealed that it provided a feeling of trust when a professional was aware of their preferences and had already prepared to perform the coping strategy.

A second way of gaining trust is that children and parents who regularly came to the outpatient department indicated that they appreciated to be known by the professional and vice versa, i.e. that they know the professional (Table 3, quote 3b). Being familiar with the professional provided a feeling of calm which made it easier to express preferences. They understood (were aware) that having exclusive involvement of professionals familiar to them is not always possible in a hospital. Nevertheless, a professional’s open and interested attitude and a positive atmosphere on the ward were still important. On the other hand, a disinterested attitude expressed by the professional was experienced as increasing feelings of unsafety, in particular by the parents.

Thirdly, children and parents stressed that professionals have to respect pre-set agreements, rituals, and boundaries of the child. Conversely, if the child’s boundaries were disregarded by the professional, it had a negative impact on the child’s trust of the whole situation concerning the procedure (Table 3, quote 3c). Almost all children who were very anxious or had been very anxious in the past had experienced a panic attack during a prior procedure during which professionals and parents did not listen to them. This caused loss of trust and high levels of fear. It took a long time to rebuild a level of trust and this often required help from other professionals.

Besides these three aspects, there were more elements that helped children to gain trust. The competence of the professional to perform the procedure and the success or failure of the procedure are an often-mentioned fourth aspect (Table 3, quote 3d). This was particularly true for needle-related procedures. Children and parents emphasized that a successful procedure instils trust. Conversely, a procedure that goes wrong undermines this trust. Some parents mentioned that then, in addition to reducing trust, a sense of control could also be lost.

### Phases in the personal process of expressing coping preferences

Despite all the different coping mechanisms and needs among children, there appeared to be a shared process regarding developing and expressing an effective preferred coping strategy.

First and foremost, each child needed the space to develop and to try out an appropriate coping strategy, which, as described earlier, is a very personal process (Table 3, quote 4a). A lot of children changed their coping strategy over time because the one they were used to no longer fitted their needs. Information about different NPIs and coping strategies and the space to try out coping strategies could help children and parents find the most suitable and personalized coping strategy. Some children and parents indicated that they would have preferred to have received this information earlier on.

Furthermore, in most of the situations, it was important that the professionals who were involved in the medical procedure were aware of the preferences of each child. Professionals need to know these preferences because they often help the child to use their preferred coping strategy. This implies that a coping strategy should be expressed by the child or parent to professionals (Table 3, quote 4b). As described earlier, it was remarkable that children and parents had different ideas and preferences about how to express and share this information with the professional. This means that for expressing coping strategies there are also preferences. Nevertheless, children and parents emphasized overall the important role professionals played in performing a coping strategy.

## Discussion

The interviews in this study demonstrated that a personal coping strategy was important for children who had experienced medical procedures. An overarching theme from all interviews was that developing and expressing a coping ritual for a medical procedure was a deeply personal process. In order to express a coping strategy, it is important that a child is given the necessary space and information to develop preferences for coping.

During the development and expression of these strategies, children experienced gaining control and feeling trust as important themes. This helped reduce children’s fear of the medical procedure they had to undergo*.* The necessity for control made it important for professionals to accommodate for the child’s coping strategy. Feelings of trust allow children and parents to express their coping strategy more easily. It was essential to parents and children that professionals were aware of personal coping strategies.

Research on the process of how children learn and express coping strategies to decrease pain and fear during medical procedures is limited. In agreement with previous studies, many children who receive painful treatments in the hospital or at home have developed a coping strategy spontaneously or with the help of their parents, these often being effective [13, 26–28]. The strategies varied between children and were used differently on different occasions [12]. Our study shows that learning effective coping strategies is a personal process. Hence, in order to accommodate the coping preferences of a child, the professional must always familiarize themselves with the personal situation of the young patient [28].

Although children could devise sufficient coping strategies on their own, children and parents had the desire to receive information about other options for effective, and possibly more optimal, coping strategies. In the interviews, this desire for more information about effective coping strategies and the possibilities available in the hospital came primarily from the parents and some older teens. Previous studies showed that younger children also feel a necessity for personalized information on better coping strategies that might help them deal with medical procedures [13, 29].

The interviews in our study revealed that the experiences, needs, and wishes in developing and expressing coping preferences evolved around two themes: gaining control and feeling trust. Children and parents sometimes found it difficult to indicate their needs and wishes in expressing coping preferences; they shared more about their experiences with medical procedures. A certain degree of control and trust was yet again prominent in these narratives. Hence, these two themes are not only essential for expressing coping preferences, but are also helpful in experiencing less anxiety about a procedure.

Although both themes were described separately in the results section to provide more clarity, the themes also seemed to be interrelated. In most interviews, both themes emerged to a greater or lesser extent. For example, if a child became very anxious about the medical procedure, it meant that self-confidence and confidence in the professionals had decreased seriously. By providing trust, empathy, and regaining control, the fear decreased and trust could be rebuilt. Expressing preferences just before a procedure was very important for some children to help them gain a feeling of control; otherwise, they did not trust that the professional knew the preferred coping strategy. However, no research that has studied the relationship between control and trust in the subjective experience of medical procedures could be found.

There has been little investigation of the need to gain control during a medical procedure. Blount et al. suggested that adults need not give too much control to the child at critical periods during the procedures [30]. Giving them too great a degree of control left them to their own devices. They distinguished decisional control, whereby the child may be offered a choice with a limitation (left arm or right arm) and behavioral control, which can give indefinite delay (when will we start). The latter situation was likely to increase distress and anxiety. Nevertheless, our study showed that if professionals did not take children’s personal boundaries into account, with subsequent loss of control, children experienced greater anxiety for a medical procedure. More research is needed on which manner (form and degree) of control helps a child feel comfortable during a medical procedure.

Much more research was present on gaining trust and developing a trustful relationship with the professional. Sheenan et al. stressed that a trusting child is likely to be less fearful, experience clinical procedures as less threatening, and is more likely to adhere to treatment [31]. Other research highlighted that when patients and professionals established mutual trust, patients demonstrated better adaptation and collaboration for improvement of health: they expressed a (greater) sense of security [32, 33]. These outcomes are consistent with our study in which children and parents indicated that it gives them a sense of calm to be known by the professional who is aware of their coping preferences.

The meaning and content of a trustful relationship between patient and professional is described in the theory of presence, developed by Baart and Klaver [34]. This theory, which is rather an approach than a method, stated that good care is tuning, effective, legitimated, feasible, and mediating [35]. According to Baart, attentive professionals take time to get to know their patients and environment deeply and strive to affirm the fundamental dignity of the person. Whilst not problem-focused, this approach may lead to person-centered problem solving. Professionals should get more training on the principles of this approach. It could inspire them to be attentive and give them a better understanding of the personal needs of children and their parents regarding medical procedures.

This study has several limitations. Children and parents were not sent a summary of their interview to verify their meaning. Furthermore, the study was conducted in a single Dutch academic hospital with a sample containing mostly well-educated, Dutch-speaking children and parents. Hence, a sample from various international hospitals may lead to different results because of cultural differences in expressing preferences.

A third limitation was the challenge to include parents of young children. It could therefore be possible that perspectives from young children were less addressed. However, interviews of parents with this target group did not provide new insights.

An important strength of this qualitative study is that to our knowledge it is the first study to describe children’s and parents’ perspectives on expressing coping preferences around medical procedures. Moreover, parents and children with different conditions and different experiences were included.

Furthermore, rigor and evaluation of trustworthiness were examined using investigator triangulation. This was attained by debating the data extraction and defining analytical themes among the researchers and within the research group.

## Conclusion

Professionals must combine clinical skills with personal attention and respect for children’s unique needs and personal boundaries in the process of searching and communicating coping preferences. Children and parents need tailored information about coping strategies to develop an optimal personal coping strategy. Training for professionals in procedural care is required that, besides technical aspects, also addresses children’s specific psychosocial care during a procedure. This gives children and parents the feelings of control and trust they need to express their preferences and to experience the least possible pain and fear during invasive medical procedures.

## Data Availability

The data that support the findings of this study are not openly available due to reasons of sensitivity and are available from the corresponding author upon reasonable request. Data are located in controlled access data storage at University Medical Center Utrecht.

## Appendix. Interview guides children and parents

### Interview guide children

1. Can you tell what procedures you have experienced (such as IV insertion, drawing blood, or inserting a tube)? What was the last time and how did you feel about it?
2. What helps you during a procedure to feel as comfortable as possible? (think about distraction techniques). And why?
3. Do you ever get asked what is for you the most comfortable way? How did you feel you could comment on that?
4. And if they don’t asked you, what then?
5. How do you know what helps you? How did you choose the most effectively way?
6. Can someone else choose for you? Who is that? Do you like that?
7. Did you know the possibilities for being distracted? How can we tell/indicate that? Would you have liked more explanation? And when (just before the procedure or a while before?)
8. What do you think about writing down your preferences? In what way would you like that (booklet, cards, app)
9. What would you say to other children, parents or staff about telling them what important is for you?
10. Are there any other events or experiences about this topic that you would like to share?

### Interview guide parents

1. What are your experiences with medical interventions your child had to undergo? How was that for you as a parent?
2. What do you do to support your child through the intervention? Would you like to do it differently?
3. Are you or your child ever asked what your child would like? How do you feel about that? Does that help your child?
4. And if not asked, what then? Do you feel space to say something that would help to communicate it anyway?
5. Did your child indicate something on his/her own or did he/she do something that helped him/her?
6. Optional: When do you say by yourself what your child wants, when do you let your child to say it?
7. What help from the professional would be useful in being able to indicate what is comfortable for your child or letting your child say what he/she is comfortable with?
8. In what way could be recorded what you and your child want? (booklet, app, something in HIX, card system)
9. What would you advise other parents or children about telling children their wishes in medical interventions that may cause anxiety and/or pain?
10. Are there any other events or experiences on this topic that you would like to share?

## Abbreviations

NPI: Non-pharmacological interventions
WMO: Wet Maatschappelijke Ondersteuning (Dutch Medical Research Involving Human Subjects Act)
UMC: University Medical Centre
